# Deep learning in veterinary medicine, an approach based on CNN to detect pulmonary abnormalities from lateral thoracic radiographs in cats

**DOI:** 10.1038/s41598-022-14993-2

**Published:** 2022-07-06

**Authors:** Léo Dumortier, Florent Guépin, Marie-Laure Delignette-Muller, Caroline Boulocher, Thomas Grenier

**Affiliations:** 1 Université de Lyon, VetAgro Sup, UPSP ICE 2021.A104, 69280 Marcy L’Etoile, France; 2 Department of Computing, Imperial College London, London, SW7 2AZ UK; 3 Data Science Institute, Imperial College London, London, SW7 2AZ UK; 4 Université de Lyon, Université Claude Bernard Lyon 1, CNRS, VetAgro Sup, UMR 5558, Laboratoire de Biométrie et Biologie Evolutive, 69280 Marcy L’Etoile, France; 5 UniLaSalle polytechnic institute, Veterinary College, campus of Rouen, 76130 Mont Saint Aignan, France; 6 Université de Lyon, INSA–Lyon, Université Claude Bernard Lyon 1, UJM-Saint Etienne, CNRS, Inserm, CREATIS UMR 5220, U1294, 69100 Lyon, France

**Keywords:** Computational models, Machine learning, Image processing

## Abstract

Thoracic radiograph (TR) is a complementary exam widely used in small animal medicine which requires a sharp analysis to take full advantage of Radiographic Pulmonary Pattern (RPP). Although promising advances have been made in deep learning for veterinary imaging, the development of a Convolutional Neural Networks (CNN) to detect specifically RPP from feline TR images has not been investigated. Here, a CNN based on ResNet50V2 and pre-trained on ImageNet is first fine-tuned on human Chest X-rays and then fine-tuned again on 500 annotated TR images from the veterinary campus of VetAgro Sup (Lyon, France). The impact of manual segmentation of TR’s intrathoracic area and enhancing contrast method on the CNN’s performances has been compared. To improve classification performances, 200 networks were trained on random shuffles of training set and validation set. A voting approach over these 200 networks trained on segmented TR images produced the best classification performances and achieved mean Accuracy, F1-Score, Specificity, Positive Predictive Value and Sensitivity of 82%, 85%, 75%, 81% and 88% respectively on the test set. Finally, the classification schemes were discussed in the light of an ensemble method of class activation maps and confirmed that the proposed approach is helpful for veterinarians.

## Introduction

Digital radiograph is a complementary exam largely used in veterinary medicine for its practicality and its quickness^1,2^. As digital radiograph, Thoracic Radiograph (TR) is especially indicated for diagnosis of intrathoracic and systemic diseases^3^. In TRs, pathological pulmonary lesions result in increasing the radiographic opacity of lungs and the radiographic appearance depends on the structures involved (interstitium, alveoli, bronchi, vessels)^3^. These characteristic features are called Radiographic Pulmonary Patterns (RPPs) and 5 types are described: the interstitial pattern, the alveolar pattern, the bronchial pattern, the vascular pattern and the nodular pattern^3^. A correct description of RPPs (localization, distribution and intensity) is crucial for diagnosis, treatment and aftercare of the animal^3,4^. However, accurate reading of a TR is challenging for veterinarians because most pulmonary diseases involve several RPPs in various pulmonary localizations, and can result in error rates concerning RPP identification on TR varying from 10% to about 50% .^3,5^.

In this context, a computer-aided-diagnosis could help veterinarians in description of RPPs. Recently, deep learning approaches based on Convolutional Neural Networks (CNN) have achieved interesting results for various diagnosis using human radiographs, such as detection of interstitial lung diseases^6^, breast cancer diagnosis^7^ or more recently for COVID-19 disease detection^8^. In veterinary medicine, studies have proved that CNN models are efficacious for classification issues with canine medical images. CNNs are able to classify superficial or deep corneal ulcers in photographs^9^, to detect diffuse degenerative hepatic diseases in ultrasound images^10^, to distinguish between meningiomas and gliomas in MR-images^11^, to detect cardiomegaly in TR images^12^ and to detect hip joints in pelvis radiographs and to classify their hip dysplasia status^13^. In TR images in particular, a CNN-based approach was more efficacious than veterinarians to evaluate ventricular and left atrial enlargement, cardiomegaly and bronchial RPP in feline and canine radiographs^5^. In feline medicine, two CNNs were evaluated for detection of thoracic abnormalities including bronchial, interstitial and alveolar RPP^14^.

However, to the best of the authors’ knowledge, no CNN has been proposed exclusively for assessing RPPs in feline TR images. In veterinary medicine, feline population is unmissable with more than 42 billions of cat owners in the US in 2020^15^, even though canine population tends to represent the majority^16^. In addition, from a deep learning perspective, studying cats enables to minimize the size and shape variation between individuals which is maximized in dogs^17,18^. For these reasons, this study focused on feline TR images.

The objective was to assess the performances of a CNN to classify feline TR images with or without RPPs and to propose an optimized framework that achieves better performances. To achieve that objective, four different pre-processings were assessed. First, the impact on the training of a manual segmentation of the intrathoracic area in TR images was assessed. Second, the use of an enhancing contrast method (ECM) was assessed. Using a voting ensemble method over 200 random shuffles of training sets and validation sets, we demonstrated that focusing the training of our CNN on a region of interest optimized its performances as described in the visual attention concept^19^, while the ECM showed no significant improvement of performances. Finally, using the Gradient-weighted Class Activation Mapping (Grad-CAM) algorithm^20^, the interpretability of the proposed CNN was discussed. Such visual explanation ensures its usefulness into everyday lives and complete the significance of the final prediction returned.

**Table 1:** Features of the database used in the study.

**Features**	Training set and validation set (normal; abormal)	Test set (normal; abnormal)
Number of cases	145; 158	20; 25
Number of cats	127; 125	20; 24
Number of TR images	230; 225	20; 25

*Number of cases* a case is defined as a veterinary visit, thus distinct cases could come from a unique cat. *Number of cats* represents the number of cats enrolled in the study. *Number of TR images* represents the number of TR images composing the sets.

**Table 2:** Number (and relative percentage) of abnormal cases showing the following radiographic findings among abnormal cases used for the training and the test.

RPP’s combination	Training set and validation set (158 cases)	Test set (25 cases)
Only one RPP	55 (35%)	8 (32%)
Only two RPPs	79 (50%)	16 (64%)
Only three RPPs	21 (13%)	1 (4%)
With four and more RPPs	3 (2%)	0
Including interstitial	126 (81%)	19 (76%)
Including bronchial	91 (61%)	15 (60%)
Including alveolar	46 (29%)	6 (24%)
Including nodular	12 (8%)	2 (8%)
Including vascular	4 (3%)	1 (4%)
Only interstitial	33 (21%)	4 (16%)
Only bronchial	14 (9%)	2 (8%)
Only alveolar	6 (4%)	1 (4%)
Only nodular	2 (1%)	1 (4%)
Only vascular	1 (<1%)	0
Only interstitial and bronchial	53 (34%)	10 (40%)
Only interstitial and alveolar	16 (10%)	2 (8%)
Only bronchial and alveolar	6 (4%)	2 (8%)
Only interstitial and nodular	2 (1%)	1 (4%)
Only interstitial and vascular	2 (1%)	0
Only bronchial and vascular	0	1 (4%)
Only interstitial, bronchial and alveolar	15 (9%)	1 (4%)
Only interstitial, bronchial and nodular	4 (3%)	0
Only interstitial, bronchial and vascular	1 (<1%)	0

**Figure 1:**
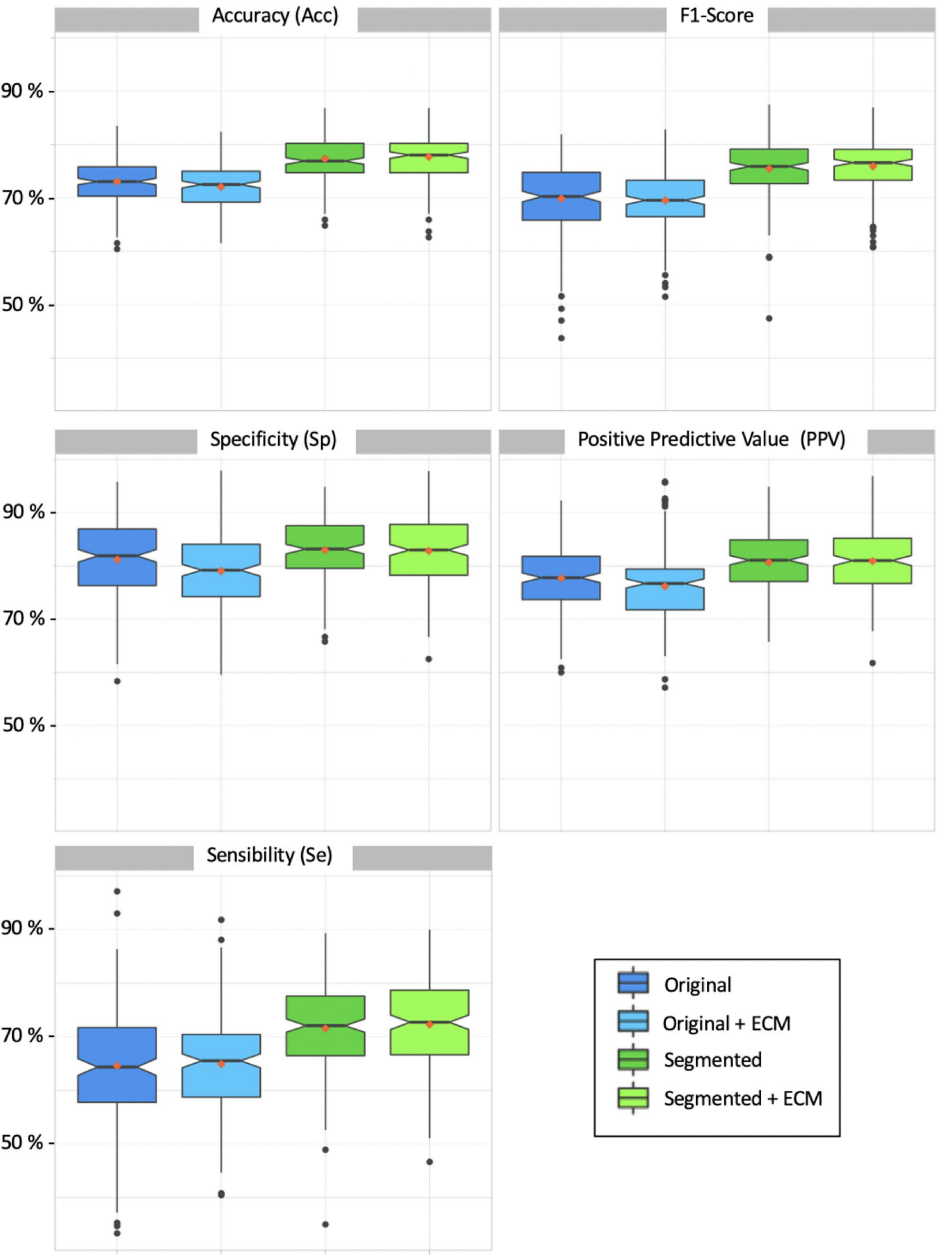
Metrics achieved over the 200 validation sets with the four pre-processings. *Notched box plots * the medians are represented by the thickest black horizontal lines framed by a notch which represents the 95% Confident Interval of the median. The red dots correspond to the means and the black dots represent the outliers which are values numerically distant from the rest of the data.

## Results

### Feline TR images database

After parsing the tabular database of all Medical Imaging Report (MIR) from the veterinary campus of VetAgro Sup, 2729 MIRs were obtained. Only MIRs which described normal or abnormal TR images are selected. TR images with extra material or extra lung disorders were excluded of the study. As cats may have been examined several times on different dates to VetAgro Sup, several MIRs could have corresponded to the same cat. Indeed, RPPs may change in the time resulting in different TR images, even though TR images came from the same cat. That is the reason why a case was defined as a single veterinary visit resulting in a unique TR exam and a unique MIR.

The database represented 348 cases, 296 cats and 500 TR images (250 normal and 250 abnormal). Training set and validation set were created with 455 TR images randomly selected corresponding to 303 cases and 252 cats. The test set was composed of the 45 remaining TR images corresponding to 45 cases and 44 cats. Details of the database are presented in Table 1. Reasons which justified the TR exams were mainly related to impairment of general condition (e.g. fatigue, anorexia, adipsia), traumatic (e.g. accident on public way, bite), cardiorespiratory signs (e.g. dyspnea, murmurs), medical follow-up and neoplastic diseases (e.g. mammary tumors, tumoral extension assessment). Supplementary Fig. S1 illustrates their frequencies.

Among the 250 abnormal TR images used in the study, 225 were allocated for the training and 25 for the test; corresponding respectively to 158 and 25 abnormal cases. Cases with a maximum of two different RPPs represented 85% and 96% of the 158 and 25 abnormal cases. Thus, the abnormal cases with strictly more than two RPPs were infrequent (< 15% and <4%, respectively for the training and the test). Cases including Interstitial RPP or including Bronchial RPP were preponderant. Interstitial RPP was described in 81% of abnormal cases used for the training and 76% of abnormal cases used for the test. Bronchial RPP was mentioned in 61% of abnormal cases used for the training and 60% of abnormal cases used for the test. The Alveolar RPP was the third most described, mentioned in 29% and 24% of abnormal cases respectively for the training and for the test. The Nodular and Vascular RPPs were in the minority, each mentioned in less than 8% of abnormal cases. All RPP combinations among the abnormal cases (e.g. only with one RPP, only with Interstitial, at least with Interstitial, only with Interstitial and Bronchial) are summarized in Table 2.

### Pretraining with human CXR images

The fine-tuning of our model with human CXR images was inspired from a Keras classification CNN that predicts presence of pneumonia^21^. A total of 5840 CXR images from the large human database were used for the fine-tuning, including 4265 CXR images of pneumonia and 1575 normal CXR images. Our model was fine-tuned with the CXR images and achieved the performances of 92% and 93% respectively for Accuracy (Acc) and F1-score on the validation set. Weights of the model obtained after this fine-tuning were used for the training with TR images.

### Pre-processing selection

From the 455 TR images used for the training, 200 training sets and 200 validation sets were built by random sampling. Performances achieved on each validation set were calculated for the four pre-processings “Original”, “Segmented”, “Original + ECM” and “Segmented + ECM”. Those performances were assessed using five metrics : Acc, F1-Score, Specificity (Sp), Positive Predictive Value (PPV) and Sensibility (Se). The distribution of the 200 values of metrics were reported in Fig. 1.

For all the metrics, a global significant difference was observed between the means achieved with respect to the four pre-processings (ANOVA p-value < 2 × $10^{-16}$ for each metric). Pair-wise t-tests with Bonferroni-Holm correction more often demonstrated significant differences between all pairs of means, except between “Segmented” and “Segmented + ECM” for all the metrics and between “Original” and “Original + ECM” for F1-score and Se. Thus, we demonstrated that segmenting TR images improves all the metrics and we demonstrated no significant improvement by applying ECM, (Fig. 1). When applied with the pre-processing “Original”, ECM even significantly decreased the means for Acc, Sp and VPP. Best global performances were achieved using the pre-processing “Segmented“ with mean values for Acc, F1-Score, Se, PPV and Se of 77%, 75%, 83%, 81% and 72% respectively (Table 3). As the pre-processing “Original” was considered as the baseline with mean values for Acc, F1-Score, Sp, PPV and Se of 73%, 70%, 81%, 78% and 65% respectively, segmenting TR images improved the mean metrics of 5.4%, 8.6%, 2.5%, 3.8% and 11% respectively.

### Classification performances with feline TR images

With the unweighted averaging voting ensemble method, the best performances were achieved with the pre-processing “Segmented” with means for Acc, F1-Score, Sp, PPV and Se respectively of 82%, 85%, 75%, 81% and 88% (Table 3). Concerning the predicted label, a TR image was classified as “Normal“ if its final prediction was <0.5 and as “Abnormal“ otherwise. The more the final prediction tends towards 0 or 1, the more the predicted label is trustworthy, considered respectively as “Normal“ or “Abnormal”.

**Figure 2:**
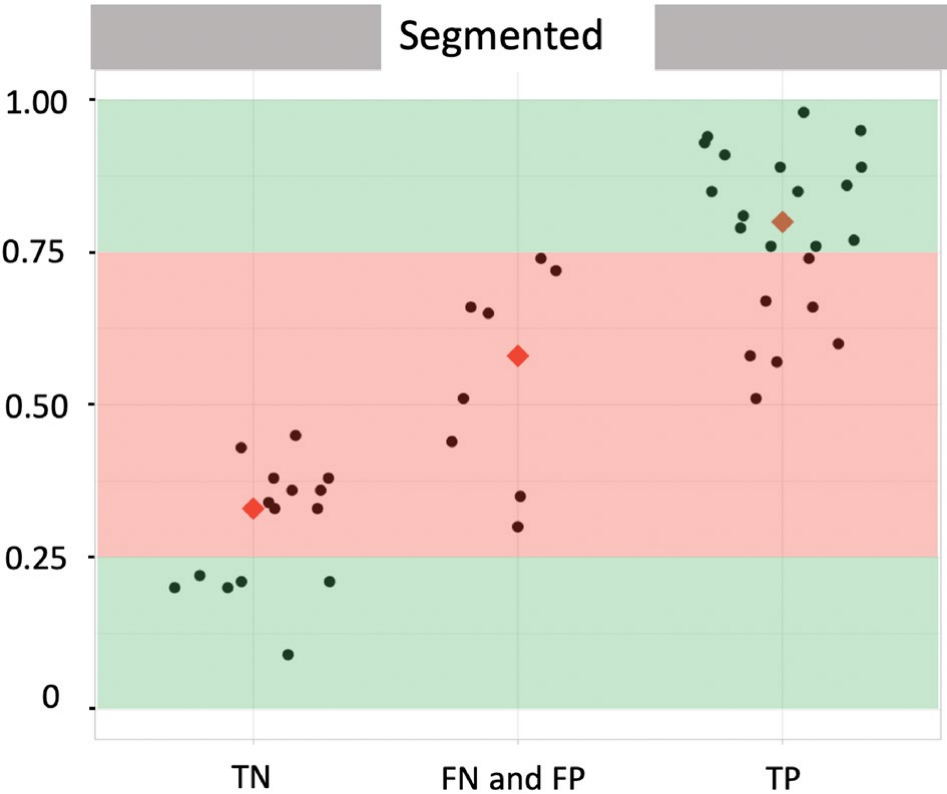
Distribution of final predictions on the test set obtained with the voting ensemble method. *Green areas* final predictions of these areas correspond exclusively to correct classifications (True Negative for final predictions lower than 0.25; True Positive for final predictions higher than 0.75). *Red area* all incorrect classifications and a part of correct classifications have their final prediction in this area. *Red diamond* The median of each of the three groups is represented by a red diamond.

For the pre-processing “Segmented”, the distribution of the final predictions were represented in Fig. 2 according to three groups : the 15 TR images correctly classified as “Normal” (True Negative), the 22 TR images correctly classified as “Abnormal” (True Positive) and the 8 misclassified TR images (False Negative and False Positive). The median predictions are 0.33, 0.58 and 0.80 respectively for True Negative, misclassifications and True Positive. All incorrect final predictions (8/8) and a part of correct final predictions (16/37) are in the range from 0.25 to 0.75. On the contrary, there are exclusively correct final predictions out of this range, i.e. lower than 0.25 and higher than 0.75. Details of the results concerning the predicted labels are presented in confusion matrices as Supplementary Table S2.

### Interpretation with averaged Grad-CAM

The activation map of the input TR image was obtained with an averaged Grad-CAM. The averaged Grad-CAM was computed from the 200 predictions used for calculating the final prediction with the voting ensemble method. The activation map for the 45 TR images of the test set enabled how far activated areas were recognized as “Abnormal”.

**Figure 3:**
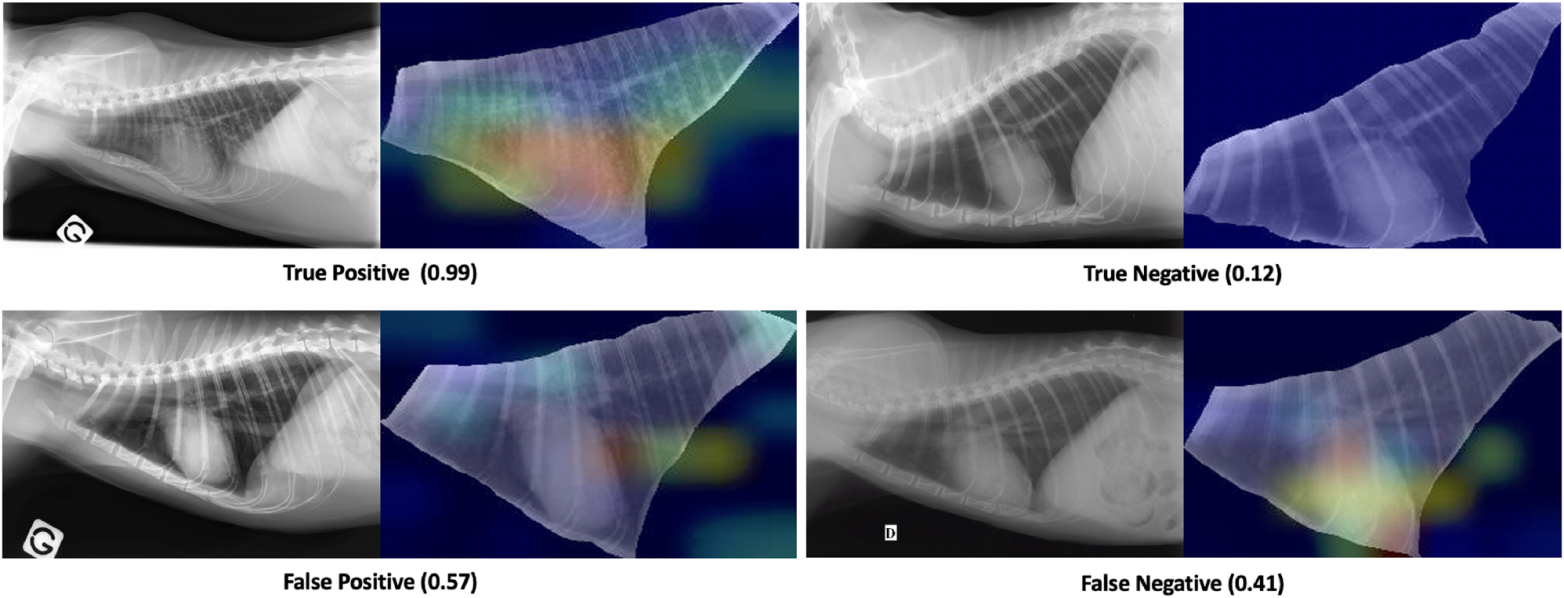
Examples of activation maps obtained with the pre-processing “Segmented“ on the test set, in comparison with the original TR image. The final prediction is indicated for each example. *True Positive* a TR image presenting a bronchial, interstitial and alveolar RPP in the caudal and accessory lobes in a context of chronic neutrophilic bronchopneumonia. *True Negative* a TR image without RPP realized after an impact with a car. *False Positive* a TR image without RPP in a context of accident on public way. *False Negative* a TR image with a focal bronchial RPP in the middle lobe, in a context of dyspnea. A zoom is applied for segmented TR images for representative purposes.

For a clear interpretation of the averaged Grad-CAM, it is important to consider the final prediction and the colors of activated areas. True Positive classifications had a final prediction higher than 0.5 associated with an activation map with warm areas. Although False Positive classifications had also a final prediction higher than 0.5, the activated areas were cool. True Negative classifications had a final prediction lower than 0.5 associated with a blue filter superimposed or with cool areas. False Negative classifications also had a final prediction lower than 0.5, but with warm activated areas. Four representative examples of classification, chosen for their relevance, are reported in Fig. 3. Exhaustive details are in free-access on GitHub (see in section “Data availability”).

The 22 TR images correctly classified as “Abnormal” were in accordance with the medical imaging findings described in their MIR, particularly for RPPs localized in the cranial, caudal and middle lobes. In addition, the cool-to-warm color scale was consistent with the severity of RPPs.

**Table 3:** Metrics achieved according to pre-processing.

Metrics	Original	Original + ECM	Segmented	Segmented + ECM
**Over 200 validation sets**				
Acc	73% (4.3%)	72% (4.4%)	77% (4.2%)	78% (4.1%)
F1-score	70% (6.8%)	70% (6.0%)	75% (5.6%)	76% (5.2%)
Sp	81% (7.4%)	79% (7.7%)	83% (6.0%)	83% (6>6%)
PPV	78% (6.2%)	76% (6.7%)	81% (5.7%)	81% (5.6%)
Se	65% (10.7%)	65% (9.5%)	72% (8.6%)	72% (8.7%)
**On test set**				
Acc	71%	69%	82%	78%
F1-score	70%	65%	85%	81%
Sp	63%	90%	75%	65%
PPV	60%	87%	81%	76%
Se	83%	52%	88%	88%

*Over 200 validation sets* the percentage represents the mean metric over the 200 validation sets with the standard deviation in parenthesis. *On test set* the percentage represents the metric on the test set with the voting ensemble method.

**Figure 4:**
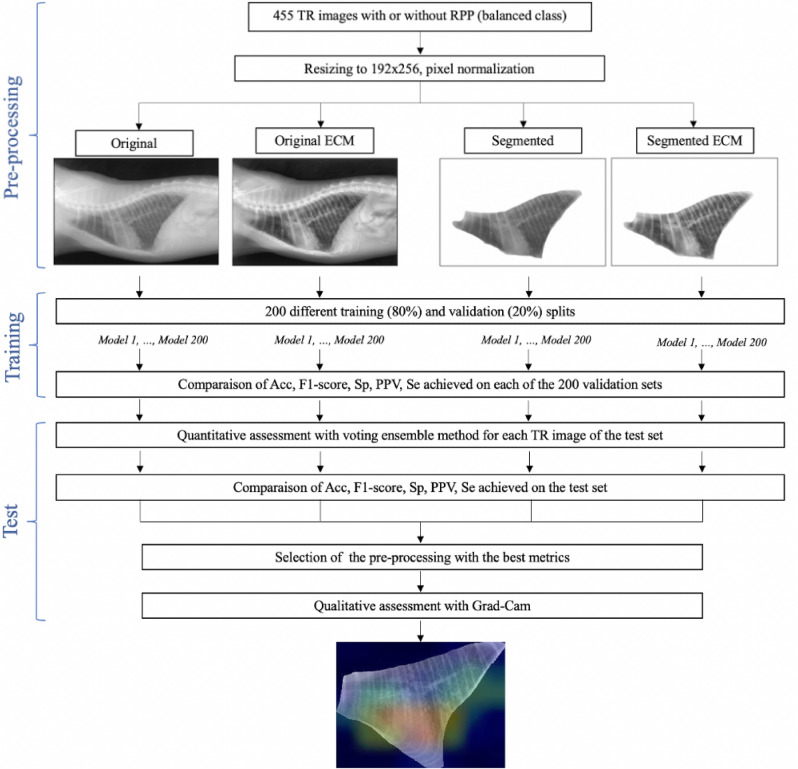
Workflow used in the study for detection of RPP in TR image. *Model 1, …, Model 200*: models fine-tuned with the 200 random shuffles splits.

**Figure 5:**
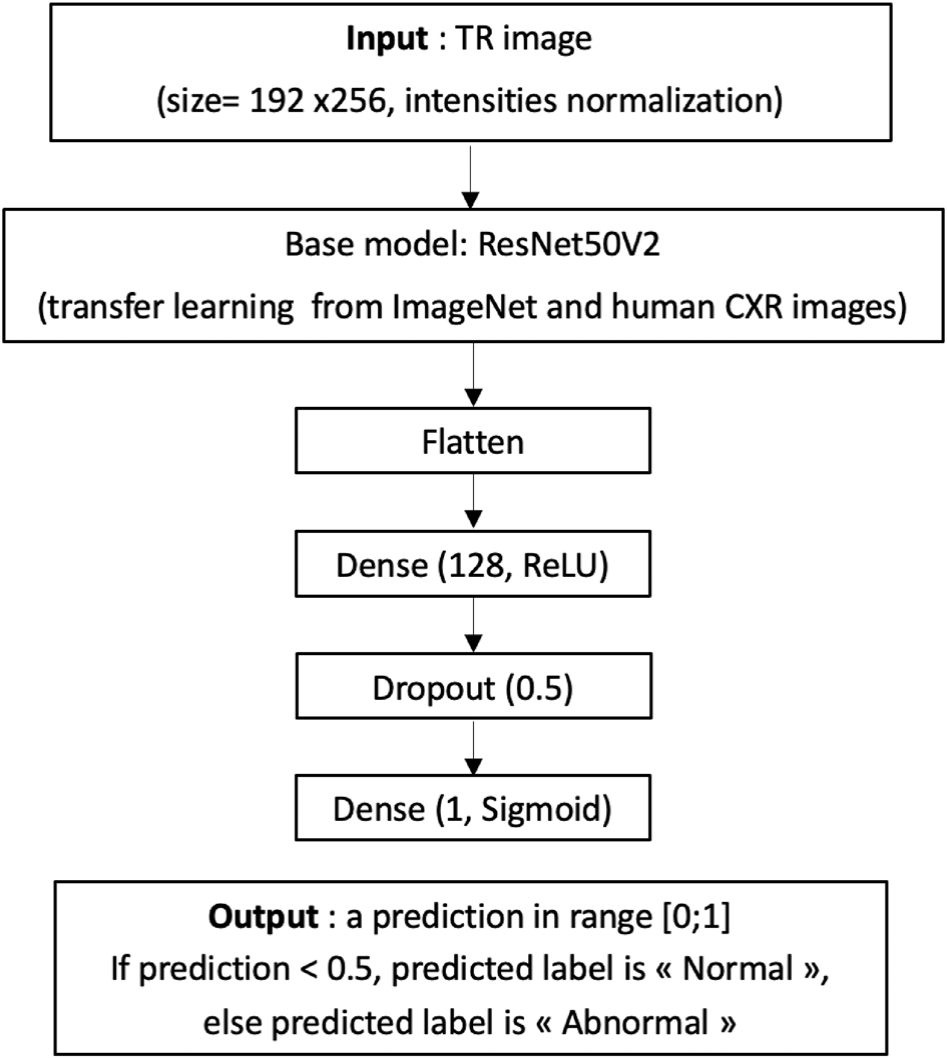
Architecture of model used in the study.

## Discussion

To the best of the author’s knowledge, this is the first study which presents a CNN-based approach to classify exclusively RPPs from feline TR images. The validity of the predicted label was assessed with an indicator (a final prediction in the range of 0 to 1) and a qualitative analysis (an averaged Grad-CAM ).

The present study was designed to overcome the major critiques of artificial intelligence in medical imaging. According to Gregory et al.^22^, these major critiques concern the lack of information on the material and method (e.g. about data, CNN or labels) and on the methodology of the analysis (e.g. about statistics, data variability).

Firstly, to overcome the database limitation problem, a complete inventory of the abnormal cases was provided. On the training sets, the validation sets and the test set, cases with interstitial RPP, bronchial RPP or alveolar RPP represent more than 85% of abnormal cases. Thus, it is expected that our approach is particularly adapted for veterinarians who suspect the presence of these frequent RPPs, but with less significance for the detection of nodular RPP and vascular RPP. Those limitations are due to the lack of TR images for these RPPs. A CNN-based approach to classify canine and feline TR images has already been described by Boissady et al., but has not concerned exclusively RPPs in cats^5^. Boissady et al. described performances of their CNN detection of RPPs: they reported error rates of about 44% for interstitial RPP, 20% for bronchial RPP, 10% for alveolar RPP and 6% for vascular RPP. Although in the present study performances of our model were not assessed according to each RPP, an error rate of 23% was achieved for all RPPs and should be lower for interstitial RPP, bronchial RPP and alveolar RPP due to the RPP’s combinations. Indeed, interstitial RPP, bronchial RPP and alveolar RPP are the three most prevalent RPP in the dataset. However, we highlight that considering each RPP independently is not more relevant than our approach which instead considered two categories : “Normal” and “Abnormal”. Indeed, Thrall et al explained that in most pulmonary diseases, more than one compartment of the lung is involved, conducting generally in more than one RPP^3^. Thus, if we want to go further than our CNN-based approach or CNN-based approaches which study RPPs independently, we should instead consider a classification according to all RPP’s combinations. But studying all RPP’s combinations with a CNN-based approach requires a much larger dataset.

Furthermore, the impacts of four pre-processings on performances were assessed. Those pre-processings have been introduced by Al-antari et al. who have proposed a CNN-based approach to detect breast cancer on mammograms consisting in segmenting first a region of interest and applying ECM^23^. To Al-antari et al., segmenting allowed a CNN to focus on a smaller region of interest and applying ECM enhanced abnormalities in surrounding tissues, therefore facilitating the diagnosis. Since such an approach has never been undertaken in veterinary medicine, the pre-processings of the present study were further investigated. To conduct our study, we assessed performances of our model with five metrics (Acc, F1-Score, Sp, PPV and Se) over 200 training sessions in order to highlight precisely the strengths and weaknesses of the four pre-processings. Moreover, the variability inherent to splitting between the training set and the validation set was considered. These 200 training sessions enabled a robust statistical analysis of results, more precise than a small k-cross validation or a single training. Hence, significant differences were obtained over all validation sets such as the pre-processing “Segmented“ improved all metrics in comparison with the baseline pre-processing “Original“. However, we obtained no significant improvement of the metrics over all validation sets with the pre-processing “ECM”, excluding our initial hypothesis about this pre-processing. According to the author’s opinion, probably that the pre-processing “ECM“ used in our study is a linear application which does not discriminate enough the difference between normal and abnormal TR images.

Above the statistical benefit for the assessment of pre-processings, the 200 random shuffles of training sets and validation sets enabled the voting ensemble method. The voting ensemble method used in our study took the unweighted average of the output prediction for all the 200 models and returned a final prediction ranged between 0 to 1. The voting ensemble method has been proved essential in the optimization of the training with a limited database. Another voting method which is called “majority voting”^24^ has also been assessed. This alternative method counted the votes of all the predicted labels from the 200 models and returned the predicted label mostly represented. Both methods were compared and the best results on the test set were achieved for the unweighted average. Moreover, the unweighted average provided a final prediction which reflected how far the predicted label was trustworthy. With our 45 TR images used for the test, the distribution of final prediction confirmed the results expected in theory : final prediction tends towards 0 for “Normal” TR image, towards 1 for “Abnormal” TR image and stays nearly 0.5 for incorrect classifications. However, we did not observe distinct ranges of final prediction between True Negative, misclassifications ans True Positive. With our test set, we observed that all final predictions lower than 0.25 truly correspond to “Normal“ and all final predictions higher than 0.75 truly correspond to “Abnormal”. The numbers 0.25 and 0.75 were determined from our 45 final predictions but could most likely change if the test set is larger. The key point of these results was that final predictions around 0 and 1 were a reliable indicator. Between 0.25 and 0.75, the veterinarian has to keep in mind that the predicted label could be incorrect.

In order to help the veterinarian for assessing TR image, especially if the final prediction is in the range of 0.25 to 0.75, we proposed a qualitative analysis of classification. With a final prediction between 0.25 and 0.75, it was impossible to know if the final prediction corresponded to a False Negative or a False positive. That is the reason why a novel approach in deep learning for veterinary medicine was developed in the present study, using averaged Grad-CAM. This new approach allowed us to visualize an activation map which represented areas recognized as “Abnormal” and how far they corresponded to the “Abnormal” predicted label. Thanks to the activation map, the veterinarian is able to differentiate a False Negative and a False Positive. Indeed, a final prediction lower than 0.5 associated with warm areas corresponded to a False Negative and a final prediction higher than 0.5 associated with cool areas corresponded to a False Positive. In addition, the veterinarian was able to confirm a correctly predicted label. A final prediction lower than 0.5 with cool areas or with a blue filter confirmed a True Negative. On the contrary, a final prediction higher than 0.5 with warm areas confirmed a True Positive.

Bearing in mind the challenge to develop a reliable CNN with a limited database, full advantages of ResNet50V2 were obtained by training on ImageNet completed by pretraining with human CXR images. The model was finally fine-tuned with TR images. The pretraining with CXR images allowed a sharper learning with human radiographs which were closer to TR images. The model without fine-tuning with CXR images was tested, but the performances were reduced.

This study has several limitations. As it is often reported, the lack of data in deep learning for medical imaging is a challenge to train a robust and scalable CNN. That is why several strategies were experimented to maximize the performances : data-augmentation, fine-tuning with CXR images, testing four pre-processings, running 200 training sessions and obtaining 200 fine-tuned models, predicting with a voting ensemble method and providing activation map. Nevertheless, enlarging at least the test set could be relevant to confirm the high performances of our model and could also create a larger training set to sharpen RPP detection. Besides, a larger dataset could permit to develop a CNN-based approach for a multi-label classification which considers all RPP’s combinations. Ventro-dorsal TR images were excluded from the study whereas they are used in routine. In the present study, lateral views were selected to keep a uniform morphology between TR images and ease the training of the model. In addition, TR images came from the veterinary campus of VetAgro Sup implicating an excellence realisation and description. So, the metrics of our model could be different in other conditions. A solution could be to use several X-ray machines, with TR images shot by several veterinarians. Another limitation of the present study was that TR images composing our database did not have major abnormalities of the pleural and mediastinal space but also of the cardiovascular system, the airways and the musculoskeletal system. Thus, our model should probably have lower performances with TR images presenting these types of abnormality. The last important limitation was the time-consuming constraint to segment manually the intrathoracic area on TR images, even if it spent less than one minute with a basic image viewer. Keeping in mind that CNN-based approaches are also elaborated to speed up workflows, we plan to elaborate an automatic CNN-based segmentation in a future work.

All in all, this is the first study in veterinary medicine which presented CNN developed to detect specifically RPPs from lateral TR images in cats, especially with a voting ensemble method associated with an averaged Grad-CAM.

The activation map combined with the final prediction really helps the assessment of the result, in particular to differentiate potential False Positive and False Negative. Thus, veterinarians might use our approach as a non-invasive complementary exam for exploring radiographically potential lung lesions. Our approach has the potential to facilitate the workflow for TR images description.

For future research, development of a multi-view CNN with veterinary TR images to consider the combination of all views appears as a very interesting question. The multi-view approach has been explored in human medical imaging for mammogram classification by Sun et al.^7^.

## Methods

### Study population

TR images were extracted from the image database of the veterinary campus of VetAgroSup, France, over the period from September 2012 to January 2020. The image database was composed of 72,567 records. In this database, each record has a Medical Imaging Report (MIR) which contained details relative to the animal (e.g. specie, age, breed), its condition (e.g. : reason of consultation, clinical sign, follow-up), the radiographic procedures (e.g. X-ray machine, projections) and the medical imaging findings. All MIRs formed a secondary tabular database, where each MIR was used to label its corresponding image from the first database. The MIR was reviewed by at least one veterinary radiologist expert (ECVDI board-certified).

### Feline TR images selection

For a same cat, sets of TR images could have been acquired on different dates and the medical imaging findings could have changed : that is why a case was considered as a couple (cat; day). For each animal, one up to three views have been realized among the following projections: left-lateral, right-lateral and ventro-dorsal. The acquisition of TR images was supervised by veterinary technicians from the diagnostic imaging unit of the veterinary campus of VetAgro Sup in accordance with their animal welfare guidelines. No direct cats were involved in this study. Two softwares were used for acquisition: ImagePilot (KONICA MINOLTA) and Console Advance (FUJIFILM CORPORATION). All MIRs and all TRs were validated by the veterinary radiologist expert. A first request on all MIRs selected those with or without description of RPP and led to the creation of two sets of cases: TR images with RPP(s) and TR images without RPP. Based on these two sets, corresponding DICOM files were extracted and then converted into JPEG files with med2image^25^. Each JPEG image and corresponding MIR were reviewed by a veterinarian to check if there is misclassification and to conserve only normal TRs or TRs with RPP(s) without any extra material (e.g. infusion line, bandage, lead shot) or extra lung disorders (e.g. diaphragmatic hernia, severe pleural effusion, pneumothorax).

### Feline TR images pre-processing

Initial TR image files size ranged from 138 to 545 KB and their image matrix size ranged from $1692 \times 1350$ to $2964 \times 2364$ pixels, with a width-height ratio from 1.2 to 1.3, depending on the size and detector density of the radiographic detector plate used during TR image acquisition. For conserving the ratio and pixel intensity range of CNN, all TR images were resized to $256 \times 192$ and normalized during pre-processing. Then, TR images were said as “Original” if no more modification was done or as “Segmented” if the intra-thoracic area was manually segmented with the image-viewer Preview (v.10.1, macOS Mojave v.10.14.6). The intra-thoracic area was defined as the radiographic part on TR image delimited dorsally by the ventral side of spine, ventrally by the dorsal side of sternum, cranially by the first ribs and caudally by the diaphragm. This segmentation was made by a unique veterinarian. Moreover, the effect of “ECM” based on Contrast Limited Adaptive Histogram Equalization for “Original“ and “Segmented“ TR images was tested^26^. These two additional image pre-processings were called respectively “Original + ECM” and “Segmented + ECM”. The manual segmentation was performed after the use of ECM. Thus, four different image pre-processings are assessed in this work. The workflow used is presented in Fig. 4.

### Model and architecture

ResNet50^27^, a well used deep learning classification architecture, recently showed the highest performance in comparison with four other CNNs for the detection of coronavirus pneumonia using CXR images^28^. Inspired by ResNet50, ResNet50V2^29^ is a modified version that performed better on ImageNet^30^, one of the hugest database composed of millions of images from hundreds of categories. That is why the model in this study was built on the model ResNet50V2. All layers above the last convolutional layer were replaced by a 4-layers block including two fully connected layers with only one neuron on the last layer for binary classification purposes. Fig. 5 details the proposed model. A binary cross-entropy loss function was used and a final output sigmoid function predicted the class. Thus, the model takes a TR image as input and returns a prediction probability in the range of 0 to 1. If the returned prediction probability is less than 0.5, the predicted label is “Normal“, else the predicted label is “Abnormal“.

Keras library on top of TensorFlow (version 2.3.0, Google) was used to implement the model. All algorithms were run on a Tesla P100 16G (NVIDIA) GPU from the computational platform of the Centre Blaise Pascal (ENS, Lyon, France). Training such a deep classification network is challenging and we proposed to use transfer learning and fine-tuning and then a data augmentation for the final model’s training.

### Transfer learning from ImageNet and human CXR images

In order to get the most out of the database of TR images on a very deep architecture such as ResNet50V2, a pre-training was performed at first on a natural image database and then a fine-tuning was run on a human radiography database. These approaches are also known as transfer learning. Transfer learning refers to storing knowledge learned from solving one problem and then using it to another related problem. In the context of classification of TR images by CNN, this corresponds to reusing the weights of a classification network trained on another database (i.e. natural color images), as initial weights for the coming training on other database (i.e. X-ray images)^31^. This strategy often produces better results after training (or fine tuning) on a new database than using random initial weights for training even for medical applications (an example using magnetic resonance imaging to evaluate positron emission tomography scans can be found in^32^). For instance a CNN model trained on ImageNet has been fine tuned for pneumonia and tuberculosis localization on radiographs^33^. Thus, transfer learning was used from ImageNet to the “Large database of Labeled Optical Coherence Tomography and Chest X-Ray”^34^ which contains hundreds of human CXR images with or without signs of pneumonia.

### Training model on feline TR images

Two sets were randomly generated from the 500 TR images: 455 TR images for the training (performed with the training set and the validation set) and 45 TR images for the test (performed with the test set). TR images used for the training and for the test represented respectively 90% and 10% of the total amount of TR images. Among the 455 TR images used for the training, 80% were allocated for the training set and 20% for the validation set. Setting of such ratios (90%/10% and 80%/ 20%) were inspired by a similar study which used a limb radiograph database for the detection of hip fractures on plain pelvic radiographs^35^.

A data augmentation strategy was applied only on the training set using the following transformations: random rotation (± 15°) random width and height shift (at max of 0.05% of total width or height), random shear (0.5) and random zoom (0.8 to 1.2). Although flipping could extend the training set, it was not used because TR images are conventionally oriented with the cat’s head and the cat’s back on the left part and on the top part of TR images respectively^36^.

Inspired from a methodological CNN-based study with ultrasound images in dogs^11^ an exponential decay learning rate schedule with an initial learning rate of 0.001 was used. The batch-size was set to 40, a dropout of 0.5 and Adam optimizer^37^ were used. The initial number of epochs was set arbitrarily to 500 although an early-stopping function was implemented to stop the training when the loss on the validation set has stopped decreasing after 25 consecutive epochs in order to avoid overfitting and reduce the training time^38^.

The model firstly fine-tuned with human CXR images was secondly fine-tuned on the 455 TR images with each of the four pre-processings (“Original”, “Original + ECM”, “Segmented”, “Segmented + ECM”). To rigorously compare the four pre-processings it was essential to run a lot of different training sessions. Indeed, it was necessary to overcome variations of training due to the distribution of the 455 TR images between the training set and the validation set^24^. That is the reason why our model was fine-tuned with 200 random distributions of the 455 TR images between the training set and the validation set. Thus, 200 different fine-tuned models were obtained and saved for each of the four pre-processings. This approach enabled a robust statistical analysis and justified the choice of the best pre-processing with a quantitative assessment over the 200 random shuffles validation sets. In the Supplementary Fig. S3, we justify the choice of the number 200 as a result of an analysis of three metrics (Accuracy, Sensitivity, Specificity) obtained on the test set, according to the number of fine-tuned models.

### Quantitative assessment and ensemble methods approach

The quantitative assessment of the 200 fine-tuned models was performed using 5 five metrics: Sensitivity (Se) also called “Recall”, Specificity (Sp), Accuracy (Acc), Positive Predictive Value (PPV) also called “Precision“ and the F1-score. Negative Predictive Value was not calculated because the TR exam is realized most of the time when abnormalities are suspected, thus PPV was preferred. Metrics were calculated such as: $\text {Se}=TP/(TP+FN)$, $\text {Sp}=TN/(TN+FP)$, $\text {Acc}=(TP+TN)/(TP+TN+FP+FN)$, $\text {PPV}=TP/(TP+FP)$ and $\text {F1-score}=TP/(TP+\frac{1}{2}(FN+FP))$. With TP, TN, FP and FN were respectively the number of True Positive, True Negative, False Positive and False Negative classifications.

These five metrics were calculated for each of the 200 validation sets for the four pre-processings. A statistical analysis of metrics’ distribution allowed to demonstrate which pre-processing achieved the best performances over 200 random shuffles validations sets. To complete the quantitative assessment, the metrics were computed also on the 45 TR images of the test set. These 45 TR images had never been used for training. For each pre-processing, the 200 fine-tuned models worked as independent classifiers of TR image, thus 200 predictions were provided for the same TR image. To take full advantage of these 200 fine-tuned models, a voting ensemble method was applied to make the final prediction. The final prediction was obtained by the unweighted averaging method which is the most common ensemble method for neural networks^24^: it consisted of considering the average of prediction of the 200 fine-tuned models. In this way, the variance of prediction from these 200 fine-tuned models was reduced and less dependent on the split between the training set and validation set^24^. The final prediction was compared to the medical imaging findings described in the MIR. For one TR, the final prediction was obtained in about 40 s.

### Qualitative assessment with an averaged Grad-CAM

To facilitate the interpretability of the final prediction an averaged Grad-CAM was applied^20^. We refer as activation map, a function that maps all the points of the input data (the TR image), to understand where the feature of interest lies. In practice, it means that it takes in input the TR image, and understand which pixels give the more information to the model for its final prediction. The averaged Grad-CAM produced an activation map of the input TR image. This activation map represented areas on the TR image which permitted the final prediction. The intensity of activation was represented by a continuum of colors from cool (blue) to warm (red) hues. For a human eye, it produces a new picture, where points of interest are highlighted.

In medical terms, these activations corresponded to “Abnormal“ areas, i.e. areas with signs of RPP. The more the color was warm, the more the activated area was recognized as a strongly abnormal area. On the contrary, an activated area in cool colors was recognized as a slightly abnormal area. Thus, with the averaged Grad-CAM the veterinarian is able to double check the final prediction with a critical and analytical eye.

### Statistic and data analysis

For each metric, a comparison of means achieved with the four pre-processings was realized with an ANOVA and followed by a pairwise t-test with the Bonferroni-Holm correction for multiple comparisons. In addition the 95% Confidence Interval (CI) of the median value for each distribution was graphically represented with notched box plots.

## Conclusion

The present study demonstrated that our approach achieved promising performances to detect RPP from lateral TR images in cats with an Acc, F1-score, PPV and Se scores greater than 80%. Segmenting TR images on a region of interest has significantly improved the CNN’s training, whereas enhancing the contrast of TR image has shown no benefit. To take full advantage of a CNN-based approach with a limited database, authors have proposed a voting ensemble method and an ensemble class activation map from 200 different training sessions.

This approach could be full of interest for veterinarians. Further studies will investigate the use of Generative Adversarial Networks to challenge the lack of medical image in veterinary medicine.

## Supplementary Information

Supplementary Information 1.

## Data Availability

The database of TR images and the algorithm used in the current study are not publicly available because they are property of the veterinary school of VetAgro Sup but authors remain available for sharing on reasonable request. However, details of results (TR images, their corresponding activation map, their final prediction, their predicted label and their label) obtained on the test set are provided in free-access on GitHub, /Latreth/Vet_public repository (https://github.com/Latreth/Vet_public/).
