# Supplementary material for: Deep learning in veterinary medicine, an approach based on CNN to detect pulmonary abnormalities from lateral thoracic radiographs in cats
Source: Sci Rep. 2022 Jul 6;12:11418. doi: 10.1038/s41598-022-14993-2 (PMC9258008; doi:10.1038/s41598-022-14993-2)
Supplement: Supplementary file 1 — Supplementary Information 1. [file 41598_2022_14993_MOESM1_ESM.pdf]

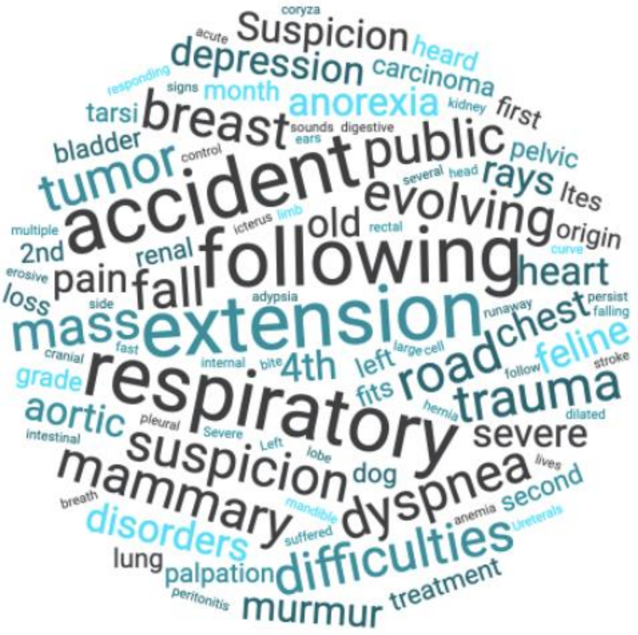

Supplementary Figure 1. Word cloud of reasons which justified TR exams in the study. **Size:** the size is proportional to the frequency of the reason; the bigger the size, the more frequent is the reason. **Colors:** colors are automatically generated for a better reading.

	Label : "Normal"	Label : "Abnormal"
Pre-processing "Original"		
**Predicted label : "Normal"**	17	3
**Predicted label : "Abnormal"**	10	15
Pre-processing "Original + ECM"		
**Predicted label : "Normal"**	18	12
**Predicted label : "Abnormal"**	2	13
Pre-processing "Segmented"		
**Predicted label : "Normal"**	15	3
**Predicted label : "Abnormal"**	5	22
Pre-processing "Segmented + ECM"		
**Predicted label : "Normal"**	13	3
**Predicted label : "Abnormal"**	7	22

Supplementary Table 2. The raw confusion matrices of classification results obtained with the voting ensemble method on the test set, according to the pre-processing.

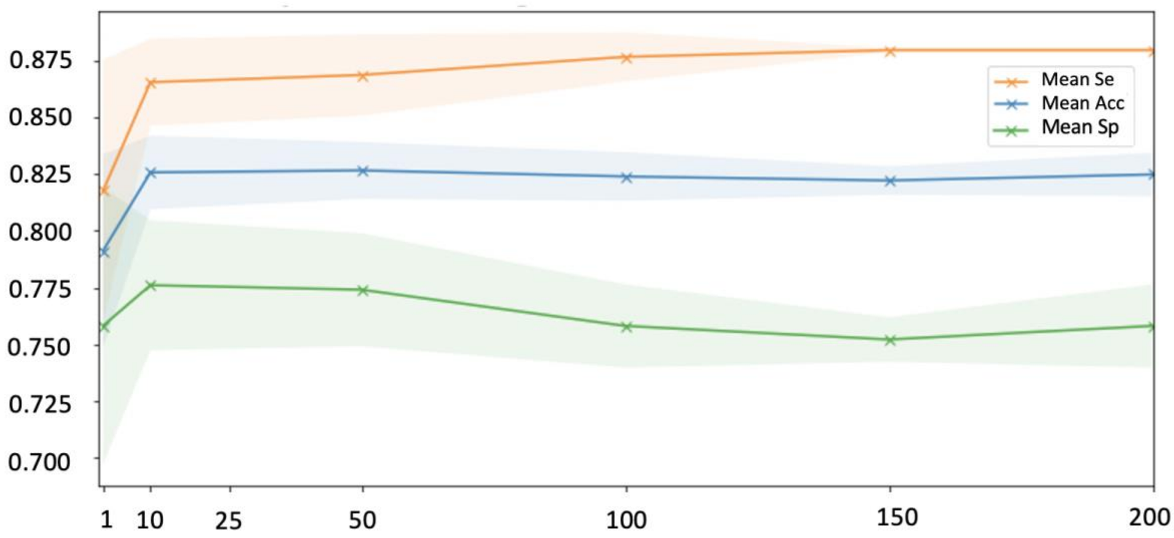

Supplementary Figure 3. Assessment of the average Accuracy (Acc), Sentivity (Se) and Specificity (Sp) and their corresponding standard deviation.

Orange, blue and green crosses : respectively value of the average Sensitivity, Accuracy and Specificity, calculated according to the number of fine-tuned models.

Orange, blue and green areas : respectively value of the standard deviation of Sensitivity, Accuracy and Specificity, calculated according to the number of fine-tuned models.
